# Quantifying the number of US men with erectile dysfunction who are potential candidates for penile prosthesis implantation

**DOI:** 10.1093/sexmed/qfad010

**Published:** 2023-04-17

**Authors:** Sirikan Rojanasarot, Abimbola O Williams, Natalie Edwards, Mohit Khera

**Affiliations:** Health Economics and Market Access, Boston Scientific, Marlborough, MA 01752, United States; Health Economics and Market Access, Boston Scientific, Marlborough, MA 01752, United States; Health Services Consulting Corporation, Boxborough, MA 01719, United States; Baylor College of Medicine, Houston, TX 77030, United States

**Keywords:** erectile dysfunction, prevalence, penile prosthesis, sexual dysfunction, census data

## Abstract

**Introduction:**

Penile prosthesis implantation (PPI) is a treatment option recommended in clinical guidelines for erectile dysfunction (ED). However, a limited number of urologists perform PPI procedures in the United States.

**Aim:**

To quantify the number of insured men with ED in the United States and project the number of potential candidates for PPI in 2022.

**Methods:**

An Excel-based disease impact model was constructed using a top-down estimation approach. The starting US male population consisted of adult men from 2022 US Census data after exclusion of age-specific mortality rates from the National Vital Statistics Reports. Men with health insurance were included in the model based on insurance status data from the US Census database. ED prevalence and ED treatment rates were obtained from administrative claims data analyses—the Merative MarketScan Commercial Database (18-64 years) and the 5% Medicare Standard Analytical Files (≥65 years)—and literature-based estimates of patient-reported ED prevalence.

**Outcomes:**

The number of men with ED in the United States and the number of potential candidates for PPI were estimated.

**Results:**

By utilizing ED prevalence based on administrative claims, an estimated 8.3% of insured men (10,302,540 estimated men [8,882,548 aged 18-64 years and 1,419,992 aged ≥65 years]) had a diagnosis of ED and sought ED care, out of 124,318,519 eligible US men aged ≥18 years in 2022. An estimated 17.1% of men with an ED diagnosis claim could benefit from PPI in 2022 (1,759,248 men aged ≥18 years). Patient self-reported ED prevalence across all ages ranged from 5.1% to 70.2%. Scenario analyses applying the patient self-reported ED prevalence range revealed the number of men in the United States who could benefit from PPI could have been higher than 1.7 million if their ED symptoms were diagnosed by health care providers.

**Clinical Implications:**

Most men with ED in the United States are undertreated, and many could benefit from PPI.

**Strengths and Limitations:**

This analysis is a US population–level estimation. However, given this study utilized a variety of assumptions, the results may vary if different model assumptions are applied.

**Conclusions:**

This disease impact model estimated that approximately 10.3 million men were diagnosed with ED by their health care providers and sought ED care in the United States in 2022. Of those, 1.7 million men could be PPI candidates and benefit from the treatment option.

## Introduction

Erectile dysfunction (ED) is a common condition affecting men that substantially and negatively affects intimate relationships, quality of life, and self-esteem.^1–4^ A prospective cross-sectional international study of 52,697 men in Brazil, China, France, Germany, Italy, Spain, the United Kingdom, and the United States between the ages of 40 and 70 years found that men with ED had significantly lower physical and mental component quality-of-life scores and significantly lower health state utilities than men without ED (all *P* < 0.001).^4^ More than half of men within the same age group experience at least partial ED, and nearly 10% have severe or complete ED.^5^ The prevalence and burden of ED increase as men age^6^; however, recent research showed a higher-than-expected prevalence of ED in young men (8% aged 20-30 years).^7^ The higher prevalence of ED in young men may be due to psychological factors such as anxiety.^7^ Men with ED are reported to have significantly higher absenteeism (7.1% vs 3.2%), presenteeism (22.5% vs 10.1%), overall work productivity impairment (24.8% vs 11.2%), and activity impairment (28.6% vs 14.5%) as compared to men without ED.^4^

Several medical and surgical treatment options are available for the treatment of ED, including oral and parenteral drugs, injectable vasodilator agents, vacuum erection devices, penile prosthesis implantation (PPI), lifestyle modifications, and psychosexual therapy. Oral phosphodiesterase-5 inhibitors (PDE5Is) are the most commonly used first-line treatment for ED; however, approximately one-third of men with ED do not respond to PDE5Is and some patients are not able to take oral medications due to medical conditions.^8^ Evidence has shown that one-third of men using PDE5Is cease use after 1 prescription and one-half cease use by 6 months.^9^ PPI is a definitive treatment option for ED, as recommended by the American Urological Association (AUA),^10^ Canadian Urological Association,^11^ British Association of Urological Surgeons,^12^ International Consultation on Sexual Medicine,^13^ Sexual Medical Society of North America, European Association of Urology,^14^ Urological Society of Australia and New Zealand,^15^ and Korean Society for Sexual Medicine and Andrology.^16^ In most cases, PPI is provided for those patients who did not respond to, did not tolerate, or were unwilling to consider other treatments. The AUA guideline states that “using the shared decision-making process as a cornerstone for care, all patients should be informed of all treatment modalities that are not contraindicated, regardless of invasiveness or irreversibility, as potential first-line treatments.”^10^ However, there are a limited number of implanters who can perform the procedure, potentially restricting access for men who may benefit from the treatment.^17^ This study aimed to estimate the number of men in the United States with ED who were seeking ED care and to identify how many men may be potential candidates for PPI as of 2022.

## Methods

### Model design and starting patient population

An Excel-based disease impact model was constructed via a top-down estimation approach. Top-down estimation captures national long-run average resource utilization or costs and provides approximate overall estimates.^18^ This is in contrast to bottom-up methods, which require local resource utilization and costs of specific inputs and capture site-level differences more appropriate for specific settings of care.^18^^,^^19^ Data from the 2022 US Census^20^ were used to determine the starting US male population (101,599,370 men aged 18-64 years and 26,727,557 men aged ≥65 years). The average age-specific mortality rates obtained from the National Vital Statistics Reports were applied to the starting population to include only the estimated men who would survive through 2022. Table 1 presents the inputs used in the disease impact model. Since this study does not involve human participants, neither institutional review board approval nor informed consent was obtained.

**Table 1:** Model inputs applied to the disease impact model.

	**Model input**		
**Parameter**	**18-64 y**	**≥65 y**	**Source**
Base case analysis			
US male population in 2022, No.	101,599,370	26,727,557	US Census Bureau^20^
Average mortality rates, %	0.4	13.4	Centers for Disease Control and Prevention^40^
US insured population, %	88.1	99.0	US Census Bureau^21^
US insured population with ED diagnosis, %	10.0	6.2	Administrative claims data analysis
Diagnosed population not receiving any ED treatment paid for by primary insurance, %	94.0	99.5	Administrative claims data analysis
US male population with moderate to severe ED, %	18.2		Rhoden et al^24^
US male population with any contraindications for penile prosthesis implantation, %	1.0		Assumption based on US penile prosthesis manufacturer instruction for use^25^^,^^26^ and American Urological Association guideline^10^ contraindications of treatable/transient infections

Abbreviation: ED, erectile dysfunction.

### Men with health insurance estimates

This model included only men who were expected to have health insurance. The proportions of men with public and private health insurance were obtained from insurance status data from the US Census database (Table 1).^21^ US Census statistics on health insurance coverage are based on information collected in the 2020 Current Population Survey Annual Social and Economic Supplements and the American Community Survey.

### Estimated number of men with ED in the United States in 2022

#### Estimated number of men with ED seeking ED care calculated from ED prevalence rates obtained from administrative claims databases

To obtain a conservative estimate of the number of men with ED, reflecting a documented diagnosis of ED by a health care provider, who are actively seeking care for their ED symptoms, this model applied the ED prevalence obtained from administrative claims databases: the Merative MarketScan Commercial Database for men aged 18 to 64 years and the 5% Medicare Standard Analytical Files (SAF) for men aged ≥65 years (Table 1). The Merative MarketScan Commercial Database provides comprehensive claims data for >263 million working individuals from >160 US employers and >40 US health plans.^22^ The 5% Medicare SAF data contains all site-of-service claims data of traditional Medicare fee-for-service beneficiaries in the United States, the majority of whom are aged >65 years. Relevant *International Classification of Diseases, Tenth Revision, Clinical Modification* diagnosis codes for ED from previously published literature,^23^ including male erectile disorder (F52.21) and male ED unspecified (N52.9), were used to identify men with ED from the databases. This conservative calculation provided the estimate for the number of men with ED diagnosed by a health care provider who were seeking care for their ED symptoms with their provider. The estimate was subsequently used to calculate the number of men who were PPI candidates and ready to consider the procedure if presented to them.

#### Estimated number of men with ED calculated from patient-reported ED prevalence rates obtained from published literature

The ED prevalence obtained from the administrative claims data analyses reflects a documented diagnosis of ED by health care providers, which is the number of men already seeking care for ED and likely underestimates the actual US prevalence of ED. Therefore, the model used patient-reported ED prevalence data obtained from a literature review we conducted in the PubMed.gov database to estimate a more realistic number of men with ED in the United States in 2022. This estimate provided the upper estimate of the range of the number of men with ED and was subsequently used to calculate the number of men who may benefit from PPI if their ED is diagnosed by a health care provider.

The literature search strategy to obtain the patient-reported ED prevalence data utilized the following search terms: “erectile dysfunction,” “prevalence,” and “United States.” The literature search was restricted to studies published between 2000 and 2022 and those published on human data and in English. The search identified 652 records that were individually reviewed to identify relevant studies reporting US prevalence rates for ED. All eligible studies underwent title and abstract screening, and any potentially relevant citation was screened via a full-text review. Reference lists of selected articles were searched for relevant data publications.

Studies were included for consideration (n = 53) if they evaluated the patient-reported prevalence of ED among a general population of US patients (ideally nationally representative), as these were deemed to be the best data sources for capturing diagnosed and undiagnosed ED. Studies were excluded if they were nonhuman or non-English, were conducted outside the United States, did not contain primary data (eg, literature reviews [systematic or narrative], meta-analyses, commentaries, editorials, or study designs), did not report clinical data (eg, laboratory data or biomechanical data), did not evaluate ED prevalence, or were small case series (n < 10 patients) or case studies. Data extracted from the most pertinent studies included the authors' names, publication year, data source/study population, methods of ED measurement, and the reported ED prevalence estimates with 95% CIs (if available) by age group (Table 2).

**Table 2:** Published patient-reported ED prevalence rates among US population (2000-2022).

**Study**	**Year**	**Data source/study population**	**ED measurement**	**Age, y**	**Prevalence (95% CI), %**
Kupelian^41^	2008	Boston Area Community Health Survey	IIEF-5	30-79	20.7
Laumann^42^	2007	Male Attitudes Regarding Sexual Health Study	MMAS	≥40	22.0 (19.4-24.6)
				40-49	9.1 (5.9-12.2)
				50-59	15.2 (11.3-19.1)
				60-69	29.4 (22.8-35.9)
				≥70	54.9 (46.9-62.8)
Derby^43^	2001	MMAS	MMAS	40-69	50.0 (45-54)
			IIEF	40-69	50.0 (42-57)
Londoño^44^	2012	Kaiser Permanente Southern California	MMAS	45-69	57.8
Ansong^45^	2000	4 rural counties in central New York State	Two questions: Have you experienced erectile dysfunction (impotence) within the past 6 mo? Have you sought treatment for erectile dysfunction (impotence)?	50-76	46.3
				50-54	26
				55-59	34.9
				60-64	46.9
				65-69	57.8
				70-76	69.4
Laumann^46^	2009	Random national sample	Two questions: Whether they had experienced erection difficulties for at least 2 mo during the previous year. Those who answered yes were then asked whether they had experienced the problem occasionally, sometimes, or frequently.	40-80	22.5 (19.6-25.7)
Selvin^47^	2007	NHANES	Question: How would you describe your ability to get and keep an erection adequate for satisfactory intercourse? Would you say that you are always able or almost always able to get and keep an erection, usually able to get and keep an erection, sometimes able to get and keep an erection, or never able to get and keep an erection?	≥20	18.4
				20-39	5.1
				40-59	14.8
				60-69	43.8
				≥70	70.2
Johannes,^48^ Feldman^5^^,^^a^	2000, 1994	MMAS	MMAS	40-49 50-59	12.4 (9.0-16.9) 29.8 (24.0-37.0)
				60-69	46.4 (36.9-58.4)
Fang^49^	2015	Boston Area Community Health Survey	IIEF-5	29.4-79.7	47.5
Rosen^50^	2004	Men’s Attitudes to Life Events and Sexuality Study	Self-reported erection difficulty	20-29	8
				30-39	11
				40-49	15
				50-59	22
				60-69	30
				70-75	37
Goldstein^51^	2020	National Health and Wellness Survey	Self-reported difficulty in achieving or maintaining an erection in the past 6 mo	40-70	46.1
Loprinzi^52^	2015	NHANES	Question: How would you describe your ability to get and keep an erection adequate for satisfactory intercourse?	50-85	53.7
Foster^53^	2013	National Health and Wellness Survey	American Urological Association–Symptom Index	≥40	24.6 for ED only and 4.9 for ED with benign prostatic hyperplasia
Shaeer^54^	2012	Global Online Sexuality Survey	IIEF-5	Mean 52.4	37.7 had various degrees of ED: mild, 19.4; mild to moderate, 7.3; moderate, 4.4; and severe, 6.6

(*Continued*)

**Table 2:** Continued

**Study**	**Year**	**Data source/study population**	**ED measurement**	**Age, y**	**Prevalence (95% CI), %**
				18-39	29.7
				40-49	23.6
				50-59	30.8
				≥60	57.4
Smith^55^	2009	California Men’s Health Study–Kaiser Permanente	Question: Many men have difficulty getting and keeping an erection that is rigid enough for satisfactory sexual activity. How would you describe your experience during the past year?	45-49	13
				50-59	24
				60-69	44
Lindau^56^	2007	National Social Life, Health, and Aging Project	Self-reported difficulty in achieving or maintaining an erection	57-85	37
Francis^57^	2007	NHANES	Self-reported ability to get and keep an erection		**Complete ED**
				Overall	8.1
				40-49	1.23
				50-59	3.65
				60-69	14.17
				70-79	24.91
				≥80	56.02
Saigal^58^	2006	NHANES	Question: How would you describe your ability to get and keep an erection adequate for satisfactory intercourse?”		**Sometimes able**
				20-29	4.7
				30-39	3.4
				40-49	7.0
				50-59	19.9
				60-69	27.0
				70-74	38.7
				≥75	30.1
Shabsigh^59^	2005	Cross-national survey on men’s health issues	Self-reported difficulty getting or keeping an erection	20-75	25
O’Leary^60^	2003	Olmsted County Study of Urinary Symptoms and Health Status Among Men	Self-reported erectile function	40-49	3
				80-89	49
Kantor^61^	2002	General medical practices in Pennsylvania	IIEF	18-40	13.0
				41-50	3.4
				51-60	28.9
				61-70	41.7
				>70	66.0
Monga^62^	2002	Community-dwelling older men	IIEF-5		**Complete, severe, moderate, mild ED**
				Overall	3, 11, 13, 24
				30-49	0, 3, 4, 12
				50-64	2, 8, 12, 32
				65-69	3, 20, 22, 30
				70-74	6, 27, 16, 27
				75-79	12, 24, 21, 29
				≥80	8, 17, 29, 38

Abbreviations: ED, erectile dysfunction; IIEF, International Index of Erectile Function; MMAS, Massachusetts Male Aging Study; NHANES, National Health and Nutrition Examination Survey; PPI, penile prosthesis implantation.

a Two publications, Johannes et al^49^ and Feldman et al,^5^ used the same patient sample to calculate ED prevalence; thus, data extraction was reported together to prevent double counting.

Self-reported ED prevalence estimates for all age groups were extracted, and the median, minimum, and maximum values were calculated. The values were then used to estimate the number of men with ED and the number of men who could benefit from PPI in the model.

### Men who received no ED treatment paid by their insurance

The model then applied the rates of men with ED who had not received ED treatment to the 2 sets of estimated numbers of men with ED calculated from administrative claims databases and published literature. The rates of men with ED but without ED treatment paid by their insurers were obtained from the same administrative claims databases (Merative MarketScan Commercial Database for men with ED aged 18 to 64 years and 5% Medicare SAF for men with ED aged ≥65 years) to calculate the number of men receiving no documented treatment during that given year. The ED treatment included PDE5Is, PPI, and other ED treatments (eg, vacuum pump, intraurethral suppositories) identified via relevant codes from previously published research.^23^

### Men who could benefit from PPI treatment estimates

For the estimation of men who were eligible for PPI, the following assumptions were made:

- Few men would pay 100% out-of-pocket for PPI if they did not have insurance; hence, these patients were not included.
- Men with moderate to severe ED (as estimated from Rhoden et al^24^; Table 1) could seek PPI after trying other ED treatments, since men with mild ED are not considered candidates for PPI.
- Only men with ED without resistant infections could be considered candidates for PPI, based on products’ instruction for use^25^^,^^26^ and AUA guideline^10^ contraindications of treatable/transient infections. It was assumed that 1% of men had a resistant infection.

It was assumed that 1% of men had a resistant infection (Table 1). The assumptions were applied to 2 sets of estimated numbers of men with ED who received no ED treatment.

## Results

### Estimated number of men with ED seeking ED care in the United States in 2022

**Figure 1:**
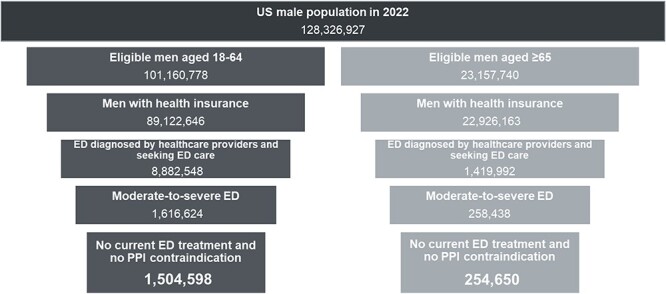
Funnel plots narrow the number of men from 2022 census data to the number of men with ED who could be PPI candidates. Data are based on ED prevalence from administrative claims databases reflecting men with ED who were diagnosed by a health care provider and seeking ED care. Abbreviations: ED, erectile dysfunction; PPI, penile prosthesis implantation.

By using the ED diagnosis data obtained from the administrative claims data analyses, an estimated 8.3% of insured men (10,302,540 estimated men [8,882,548 aged 18-64 and 1,419,992 aged ≥65 years]) were diagnosed with ED by health care providers from the 124,318,519 eligible US men aged ≥18 years in 2022 (Figure 1).

### Estimated number of men with ED calculated from patient-reported ED prevalence rates obtained from published literature

The literature review to obtain the patient-reported prevalence of ED among the general population from 2000 to 2022 identified 22 eligible publications that reported data on patient-reported ED prevalence. The ED prevalence from the included studies was assessed through different methods: International Index of Erectile Function (6 publications, 27.3%), the Massachusetts Male Aging Study–derived questionnaire (4 publications, 18.2%), and other patient self-reported questionnaires (12 publications, 54.5%). The range of patient self-reported ED prevalence across ages from 22 studies was from 3.0% to 70.2%, with a median of 27.0% (Table 2). Based on the median ED prevalence, the number of men with ED in the United States could be as high as 30,333,668.

### Estimated number of men who could benefit from PPI treatment

Figure 1 shows a funnel plot that demonstrates each step to narrow down the number of men from the starting population to the number of men who could benefit from PPI treatment. The base case estimate according to administrative claims data for the rates of men with ED without ED treatment and from published literature for the proportion with moderate to severe ED revealed that 17.1% of men aged ≥18 years with an ED diagnosis could benefit from PPI (1,759,248 men in the United States). The estimated number of men with ED who could benefit from PPI, by age group, is 1,504,598 men aged 18 to 64 years and 254 ,650 men aged ≥65 years (Figure 1). By applying the patient self-reported ED prevalence range to the scenario analyses, as many as 13,436,363 men in the United States with ED could benefit from PPI if their ED condition was clinically diagnosed by a health care provider.

## Discussion

The results of this study illustrate a high number of men with ED who are potential candidates for PPI. An estimated 8.3% of insured men in the United States (10,302,540) had a diagnosis of ED from 124,318,519 eligible US men aged ≥18 years in 2022. At least 1.7 million men are PPI candidates, and as many as 13.4 million US men can benefit from PPI if their self-reported ED is clinically diagnosed by a health care provider.

Penile prosthesis is a well-known alternative treatment option for ED with high satisfaction rates.^27–30^ IPPs provide a durable treatment, with devices functioning for up to 20 years.^31^ One publication showed that patients with ED who underwent penile implant surgery had significantly better erectile function and treatment satisfaction rates than patients who received PDE5Is.^32^ Unfortunately, PPI accessibility could be a barrier to treatment; only 4 out of 100 practicing urologists are trained to perform PPI in the United States.^17^ Ensuring sufficient penile prosthesis implanters could mitigate the potential physical, emotional, and social burden of untreated ED.

Large multicenter clinical trials have demonstrated the efficacy and tolerability of PDE5Is in ED among patients with varying etiologies and across a broad range of severity and age.^33^ However, approximately 30% to 35% of patients prescribed PDE5Is fail to respond to therapy, and an equally large proportion stops therapy for other reasons. The PDE5I prescription renewal rate has been estimated to be 62% at 3 to 4 months and 30% at 6 to 12 months.^34^ Reasons for failure of PDE5I treatment may include severe ED at presentation, worsening of endothelial dysfunction and progression of penile atherosclerosis, ED after radical prostatectomy, unrecognized hypogonadism, inadequate patient education and incorrect drug usage, and psychosocial factors.^33^ Regardless of the reason, medical therapy is evidently inadequate for a large proportion of patients with ED.

When ED goes untreated, psychological problems may ensue, such as depression, loss of self-esteem, feelings of worthlessness, interpersonal relationship strain, and cognitive issues.^35^^,^^36^ With respect to the association between ED and depression, one study demonstrated that treatment for ED is protective against the development of major depressive disorder within 3 years.^37^

Psychological impairment may also lead to missed workdays, presenteeism, and activity impairment.^4^^,^^38^ It is imperative that the urologic community understand the importance of addressing the scarcity of trained and practicing surgeons to ensure equitable access for patients.

The strengths of this study include the use of multiple data sources, such as nationwide population-level databases (census data, National Vital Statistics Reports data), two large administrative claims databases (Merative MarketScan Commercial Database and the 5% Medicare SAF), and a synthesis of information on patient-reported prevalence in a practice setting. This approach provided a large national sample, ensured comprehensiveness of our data, and increased the external validity of our findings to the United States. Second, an exhaustive analysis of the population-based data was done to characterize the US ED population by age (18-64 and ≥65 years) and can be valuable for clinical and policy decision-making. Third, implementing a disease impact model allowed for an estimation of the ED population size of the diagnosed and undiagnosed, which further helps with informing national policies and treatment recommendations.

There are several limitations to our study, many of which are inherent to all decision-analytic modeling studies. Models represent a simplification of disease and treatment pathways and combine data inputs from multiple sources. Model inputs from the published literature may be out-of-date given the evolving and aging population dynamics, changes to clinical care, and technological innovation. However, we expect that the estimates that we obtained are conservative and that the true number of men with ED and number of men potentially eligible for PPI are even higher. ED treatments may not have all been captured in the claims data and are likely underestimated (e.g., medications purchased out-of-pocket or online without a prescription, compounding pharmacy treatments, traditional therapies such as herbals, or lifestyle treatments). In addition, inferences cannot be drawn on the men whose other treatments failed outside of the claims data period. The ED prevalence obtained from the administrative claims data analyses reflects a documented diagnosis of ED, which is the number of men already seeking and receiving care for ED and underestimates the actual US prevalence of ED. Hence, to ensure that we were capturing men who may not have had access to ED care, we also utilized published estimates of patient-reported ED prevalence. Finally, the results of this modeling evaluation reflect US patients with public or private insurance, and results may not be generalizable to patients without health insurance, patients with Veterans Affairs or Tricare health insurance, or patients in which clinical practice and reimbursement structure, health care accessibility, and treatment accessibility may differ. Evidence suggests that military service members and veterans may be at increased risk for ED.^39^; however, this study did not incorporate Veterans Affairs and Tricare data.

## Conclusions

This disease impact model approximated that 10.3 million men were diagnosed with ED by their health care providers and sought ED care in the United States in 2022. An estimated 17.1% of these men (1.7 million) are PPI candidates, and ensuring sufficient penile prosthesis implanters could mitigate the physical, emotional, and social burden of ED.
